# Effect of Limited Enzymatic Hydrolysis on Structural and Functional Properties of *Elaeagnus mollis* Oil Meal Protein

**DOI:** 10.3390/foods11213393

**Published:** 2022-10-27

**Authors:** Caixia Guo, Xiaoyu Zhao, Yukun Yang, Meiping Li, Ligang Yu

**Affiliations:** School of Life Science, Shanxi University, Taiyuan 030006, China

**Keywords:** *Elaeagnus mollis* oil meal protein, limited enzymatic hydrolysis, degree of hydrolysis, structural properties, functional properties

## Abstract

*Elaeagnus mollis* oil (EMO) meal, a by-product of oil production with plentiful protein, is considered a cheap and good quality source of plant protein for use in the food industry. In this study, the influence of limited enzymatic hydrolysis of EMO meal protein on the structure, solubility, foaming and emulsifying capacities was investigated in detail. The hydrolysates with different DH values (5, 10, 15, and 17) were obtained by controlling the time of enzymatic hydrolysis with alcalase. The results showed that enzymatic hydrolysis decreased molecular weight and increased flexibility and surface hydrophobicity. At the given range of pH and concentration of NaCl, the solubility, foaming and emulsifying capacities of hydrolysates were significantly improved, especially in the area of isoelectric point, and increased with the increase of DH. It was also found that the hydrolysate with DH10 had better foaming and emulsifying stability. In general, appropriate enzymatic hydrolysis could improve the functional properties in favor of their potential use as food ingredients.

## 1. Introduction

Proteins with good nutritional and functional properties has always been a vital component of food formulas, such as solubility, foamability, and emulsifiability [1]. In recent years, plant proteins have attracted people’s attention due to their low-cost and environmental friendliness. However, the poor functional properties of plant proteins, especially low foaming and emulsifying properties, restrict its use in the food industry. Therefore, improving the functional properties of plant proteins have become a hot research topic worldwide.

At present, a variety of physical, chemical and enzymatic methods have been employed to improve the functional capacities and nutritional value of plant proteins [2,3]. Among them, enzymatic treatment can enhance the functional properties of natural proteins by transforming them into peptides without affecting their nutritional values [4]. However, small peptides cannot form the protein networks required for functional properties [5]. Therefore, the control of protein hydrolysis is vital for improving the functional characteristics of the final hydrolysis products. The extent of protein hydrolysis can be assessed by the number of cleaved peptide bonds through investigating the degree of hydrolysis (DH). García Arteaga et al. (2020) found that the molecular weight of protein was decreased with the increase of DH, and morehydrophobic groups became exposed, which resulted in increased protein solubility in all hydrolysates [6]. Furthermore, the foaming and emulsifying capacities were improved by proper enzymatic hydrolysis. Some studies have also proved that the functional properties of limited enzymatic hydrolysis of protein obtained from rice glutelin, lentil protein isolate, and peanut protein isolate can be greatly improved by controlling DH [7,8,9].

*Elaeagnus mollis* Diels, which belongs to the *Elaeagnaceae* family, is mainly distributed in western China at an altitude of 800–1500 m, and is known as a precious woody oil crop [10]. *Elaeagnus mollis* oil (EMO) meal, a by-product of oil production with plentiful protein, has been considered a cheap and good quality source of plant protein for the food industry. Currently, it is often used as a feed or discarded, resulting in under-utilization and waste. Compared with traditional processing technology, the EMO meal obtained by oiling using supercritical carbon dioxide fluid extraction is mild and safe. In this process, organic solvents are entirely avoided, and protein in EMO meal is well preserved and not damaged, which makes it a promising source of protein. Recently, some researchers have explored the extraction of EMO meal protein and the functional properties of protein isolate, and only the simultaneous study was focused on improving the functional properties for food application. Therefore, the objective of this study is to improve the functional properties of EMO meal protein by controlling enzymatic hydrolysis. We compared the effects of various degrees of hydrolysis on the distribution of molecular weight, surface hydrophobicity, conformation and functional properties (solubility, foamability and emulsifiability). Furthermore, the influence of various environmental factors (pH and NaCl concentration) on the functional properties of hydrolysates were examined to evaluate which condition could be effectively used as protein ingredients in foods.

## 2. Materials and Methods

### 2.1. Materials

*Elaeagnus mollis* oil (EMO) meal, a by-product of EMO extraction by utilizing supercritical carbon dioxide extraction technology, was provided by Shanxi Qierkang Samara Biological Products Co., Ltd. (Taiyuan, China). The EMO meal had 5.27 g water/100 g, 49.43 g protein/100 g, 22.3 g fat/100 g, and 3.32 g ash/100 g sample. Bovine serum albumin (BSA), alcalase (200,000 IU/g) and Coomassie blue G-250 were purchased from Beijing Solarbio Science &Technology Co., Ltd. (Beijing, China). Tris (hydroxymethyl) aminomethane (Tris), *β*-mercaptoethanol (*β*-Me), sodium dodecyl sulfate (SDS) and 8-aniline-1-naphthalenesulfonic acid (ANS) were supplied by Sinopharm Chemical Reagent Co., Ltd. (Shanghai, China). All the chemicals used in this experiment were of analytical grade.

### 2.2. Preparation of EMO Meal Protein Isolates

100 g of EMO meal was mixed with alkaline water (pH 11.0) to give an extraction ratio of 1:75 (*w*/*v*), and the dispersion was stirred continuously for 55 min at 55 °C. Then, the mixture was centrifuged at 4000 rpm for 15 min to collect the supernatant, and the pH of the supernatant was adjusted to 4.5 using 0.5 mol/L HCl to facilitate the precipitation of proteins. Centrifugation was used to recover the protein isolates at 4500 rpm for 15 min and then the product was freeze-dried into powder for further use.

### 2.3. Preparation of EMO Meal Protein Hydrolysates

Based on preliminary experiments, the enzymatic hydrolysates were prepared by hydrolyzing EMO meal protein via alcalase at an optimal enzyme/substrate ratio. 4 g of EMO meal protein was added into 100 mL distilled water and stirred for 20 min, then 0.2 g alcalase was added after temperature and pH adjustments (55 °C, pH = 11). The protein hydrolysates with different DH values of 5, 10, 15, and 17 were prepared by terminating the reaction at the selected time intervals (30, 50, 110, and 240 min), and the hydrolysis process was stopped by heating at 95 °C for 10 min. Subsequently, the insoluble protein was removed by centrifuging at 4500 rpm for 15 min, and the supernatant of EMO meal protein hydrolysates was freeze-dried and then stored at a temperature of −20 °C. 

### 2.4. Estimation of the Degree of Hydrolysis (DH)

DH describes the number of peptide bounds that are hydrolyzed compared to the number of peptide bonds per unit weight (h_tot_). This value was determined via the pH-stat method [11] and is given in the following equation:

$$\mathrm{DH}=\frac{B\times N_{B}}{\alpha \times h_{\mathrm{tot}}\times M_{B}}$$

where B is the volume of base consumed (mL), N_B_ is the molarity of the base used, M_B_ is the mass of the hydrolyzed protein (g), h_tot_ is the theoretical overall number of peptide bonds present in the protein substrate (7.75 mmol/g protein), and *α* is the average degree of dissociation. The value of *α* was calculated by the equation:

$$\alpha =\frac{10^{\mathrm{pH}-\mathrm{pKa}}}{1+10^{\mathrm{pH}-\mathrm{pKa}}}$$

where pH represents the value at which the enzymatic hydrolysis occurred, pKa is the average pKa for the protein’s -NH^3+^ groups, calculated at 7.0.

### 2.5. Structural Properties of Hydrolysates

#### 2.5.1. Sodium Dodecyl Sulfide Polyacrylamide Gel Electrophoresis (SDS-PAGE)

SDS-PAGE was conducted on 12% separating gel and 5% stacking gel in a MINI DYCZ-24 DN Electrophoresis System (Liuyi Biotechnology, Beijing, China). 5 mg of hydrolysate was dispersed in 1 mL of sample buffer (Tris, 10% SDS, 50% glycerol, with or without 5% *β*-Me, and 0.1% bromophenol blue). The mixture was heated in a water bath at 95 °C for 5 min, and then centrifuged at 12,000 rpm for 2 min. The samples and markers were rapidly absorbed into their respective orbits, and electrophoresis was conducted at a constant voltage of 80 V. Finally, Coomassie brilliant blue G-250 was used to stain the gels for 30 min and then they were destained using 20% methanol and 10% acetic acid solution for 6 h. The molecular weight of the hydrolysate was evaluated by comparing its electrophoretic mobility to that of the marker.

#### 2.5.2. Analysis of Sulfhydryl and Disulfide Contents

The content of the sulfhydryl group (SHF), the total sulfhydryl (SHT) group, or sulfhydryl and disulfide content (S-S) of the protein hydrolysates was evaluated by using the 5, 5′-dithiobis (2-nitrobenzoic acid) (DNTB) reagent relate to the methods previously reported by Zhang and Lu (2015) with appropriate modifications [12]. 75 mg of samples were dispersed in 10 mL phosphate buffer (0.1 M at pH = 8.0) consisting of 1% (*w*/*v*) SDS and 1 mM EDTA. After stirring for 30 min, the mixture was centrifuged at 4000 rpm for 15 min. The supernatant’s protein content was determined by the Lowry method [13].

For the SHF determination, 1.5 mL of phosphate buffer (0.1 M at pH = 8.0) including 1% (*w*/*v*) SDS and 1 mM EDTA as well as 0.5 mL DNTB (pH = 8.0) were mixed with 1.5 mL of the sample solution. 0.5 mL of distilled water was used as a blank instead of DNTB. The mixture was vortexed for 30 s and then reacted for 1 h at 25 °C. Finally, the solution was centrifuged at 4000 rpm for 15 min, and the absorbance of the supernatant was measured at 412 nm. The concentration of SHF was calculated according to the following formula:

$$\mathrm{SHF}/\left(\mu \mathrm{mol}/g\right)=\frac{73.53\times A_{412\mathrm{nm}}\times D}{C}$$

where 73.53 = 10^6^/(1.36 × 10^4^), 1.36 × 10^4^ represents the extinction coefficient for the thiolate chromogen, A_412nm_ is the absorbance of the solution at 412 nm, C is the protein content of the sample (mg/mL) and D is the dilution factor.

For the SHT determination, 1 mL of sample solution was mixed with 0.05 mL *β*-Me and 2 mL of 0.1 M phosphate buffer (pH = 8.0). The buffer contained 8 M urea and 5 M guanidine hydrochloride. Then, this mixture was vortexed and incubated for 1 h at 25 °C. At the end of the reaction, 10 mL of 12% (*w*/*v*) TCA was promptly added to the mixture, and incubated for 1 h at 25 °C, then centrifuged at 4000 rpm for 15 min. The supernatant was poured out and the precipitate was repeatedly dissolved in 10 mL of 12% (*w*/*v*) TCA, and the reaction was repeated twice as described above to eliminate the *β*-Me. After that, the residue was dispersed in 5 mL phosphate buffer (0.1 M at pH = 8.0), and 0.4 mL of DNTB reagent was added into the mixture. The mixture was stirred and reacted for 1 h at 25 °C, and then centrifuged at 4000 rpm for 30 min. 0.5 mL of distilled water was used as a blank instead of DNTB, and the supernatant’s absorbance was measured at 412 nm. The SHT content was calculated according to the following formula:

$$\mathrm{SHT}/\left(\mu \mathrm{mol}/g\right)=\frac{73.53\times A_{412\mathrm{nm}}\times D}{C}$$

where all indexes in the formula have the same meaning as those in SHF expression.

S-S was calculated according to the following formula:

$$S\text{-}S/\left(\mu \mathrm{mol}/g\right)=\frac{\mathrm{SHT}-\mathrm{SHF}}{2}$$

#### 2.5.3. Surface Hydrophobicity

The surface hydrophobicity of the protein hydrolysates was determined according to the method of Li et al. (2018) with appropriate modification [14]. In this test, ANS served as a hydrophobic fluorescent probe. The samples with concentrations of 0.05–0.25 mg/mL were prepared by using 0.01 M phosphate buffer (pH = 7.0). 4 mL of sample solution was mixed with 20 μL of 8 mM ANS, and the mixture without the sample solution was used as a blank. All samples were mixed and incubated darkly for 15 min at room temperature. The fluorescence intensity of each sample was tested in a LS-55 fluorescence spectrophotometer (PerkinElmer, Kumamoto, Japan) with excitation and emission wavelengths of 390 nm and 497 nm, respectively. Meanwhile, the excitation and emission slit widths were fixed at 5 nm. The initial slope of fluorescence intensity and sample concentration (mg/mL) was estimated via the linear regression analysis and subsequently used as an index of sample surface hydrophobicity.

#### 2.5.4. Intrinsic Fluorescence and Ultra-Violet (UV) Spectroscopy

The fluorescence emission spectrometric determination of the protein hydrolysates was acquired according to the previously reported method of López et al., 2018 with appropriate modification [15]. A sample solution of 0.5 mg/mL was prepared with 0.01 M phosphate buffer at pH 7.0. The fluorescent intensity (FI) was given as a function of emission wavelength. Therefore, the emission spectrum of the sample solution ranging from 300 nm to 500 nm was obtained by fixing the excitation wavelength at 280 nm and keeping the slit width of 5 nm.

The sample was dispersed in 0.01 M phosphate buffer (pH = 8.0) to provide a final concentration of 1 mg/mL. The sample solution was scanned in the wavelength range of 250–400 nm by using a UV-2550 UV spectrophotometer (Shimadzu, Kyoto, Japan).

#### 2.5.5. Fourier-Transform Infrared (FTIR) Spectroscopy

The samples were mixed with KBr in a ratio of 1:100, ground into a fine powder and pressed into sheets. A KBr blank without sample was prepared as the control. The infrared spectroscopic scanning of the sample was performed using a Nicolet-380 Fourier-transform infrared spectrophotometer (Thermo Fisher Scientific, Waltham, MA USA) from 400–4000 cm^−1^. The resolution of the scan was set as 4 cm^−1^, and the test was conducted 64 times.

### 2.6. Functional Properties

#### 2.6.1. Solubility

The solubility of the samples was determined based on the method of Li et al. (2018) with some modifications [14]. 25 mg of protein hydrolysate was dispersed in 100 mL distilled water, and adjusted to the preferred pH (ranging from 2 to 12) and NaCl concentration at pH 7 (ranging from 0 to 1 mol/L). The liquid was constantly stirred at room temperature for 30 min. After stirring, the dispersion was centrifuged at 4000 rpm for 15 min, and the content of supernatant’s protein was analyzed. The solubility of protein was calculated according to the following formula:

$$\mathrm{Solubility}/\left(\%\right)=\frac{\mathrm{Protein}\;\mathrm{content}\;\mathrm{of}\;\mathrm{the}\;\mathrm{supernatant}}{\mathrm{Protein}\;\mathrm{content}\;\mathrm{of}\;\mathrm{the}\;\mathrm{sample}}\times 100\%$$

#### 2.6.2. Water and Oil-Holding Capacities

The water holding capacity (WHC) or oil holding capacity (OHC) of the samples was determined based on the method described previously with appropriate modifications [14]. 1 g of protein hydrolysate was mixed with 10 mL of soybean oil or distilled water, and vortexed for 30 s. The mixture was placed at room temperature for 30 min, and was then centrifuged at 4500 rpm for 15 min. The supernatant was poured out, and the precipitate was weighed. WAC and OHC were given as the weight of water or oil derived per gram of the protein hydrolysate and calculated according to the following formula:

$$\mathrm{WAC}/\left(g/g\right)=\frac{m_{1}-m_{2}}{m}$$

$$\mathrm{OHC}/\left(g/g\right)=\frac{M_{2}-M_{1}}{M}$$

where, m is weight of the protein hydrolysate, m_1_ is the weight of centrifugal tube and sample, m_2_ is the weight of centrifugal tube and precipitation, M is the weight of the protein hydrolysate, M_1_ is the weight of centrifugal tube and sample before adding soybean oil, M_2_ is the weight of centrifugal tube and sample after oil was absorbed.

#### 2.6.3. Determination of Foaming Capacity and Foaming Stability

The foaming properties were found according to the method described previously by Du et al. (2018) with some revisions [16]. 1 g of protein hydrolysate was dispersed in 100 mL distilled water with different pH values (3.0–11.0) or with different concentrations of NaCl solution (0.2–1 mol/L). The sample solution was then homogenized at 10,000 rpm for 2 min. After homogenization, the volume was recorded at 0 min and 30 min using a 100 mL graduated cylinder. The foaming capacity was found by testing the volume of foam at 0 min. The foaming stability was obtained by measuring the remaining volume of foam after homogenization for 10 min. Foaming capacity (FC) and foaming stability (FS) were calculated as follows:

$$\mathrm{FS}/\left(\%\right)=\frac{V_{2}-V_{0}}{V_{1}-V_{0}}\times 100\%$$

$$\mathrm{FC}/\left(\%\right)=\frac{V_{1}{-V}_{0}}{V_{0}}\times 100\%$$

where V_0_ is the volume of the solution prior to homogenization, V_1_ is the solution volume after homogenization for 0 min, and V_2_ is the solution volume after homogenization for 10 min, respectively.

#### 2.6.4. Determination of Emulsifying Capacity and Emulsifying Stability

Emulsifying properties were examined using the procedure of Jain and Anal (2016) with appropriate modifications [17]. 1 g of protein hydrolysate was dissolved in distilled water at different pH values (3–11) or with different NaCl concentrations (0.2–1 mol/L). The sample solution was mixed with the soybean oil at a ratio of 3:1. Then the mixture was homogenized at 10,000 rpm for 2 min. After homogenization, 100 μL of emulsion was pipetted out from the bottom at 0 and 10 min, and diluted with 5 mL of 0.1% SDS solution. The absorbance of the diluent was determined at 500 nm using the ultraviolet spectrum. Emulsifying activity index (EAI) and emulsion stability index (ESI) were calculated using the following equation:

$$\mathrm{EAI}/\left(m^{2}/g\right)=\frac{2\times 2.303\times A_{0}}{0.25\times m}$$

$$\mathrm{ESI}/\left(\min\right)=\frac{A_{0}\times \Delta t}{A_{0}-A_{10}}$$

where, m is the protein hydrolysate weight, A_0_ is the emulsion absorbance at 0 min, A_10_ is the emulsion absorbance at 10 min, and ∆t refers to 10 min.

### 2.7. Statistical Analyses

All experimental data were collected in triplicate, and presented as means ± standard deviation (SD). Data analysis was performed using IBM SPSS 19.0 software. The differences between the mean values of samples were considered significant at *p* < 0.05.

## 3. Results and Discussion

### 3.1. SDS-PAGE Analysis

**Figure 1:**
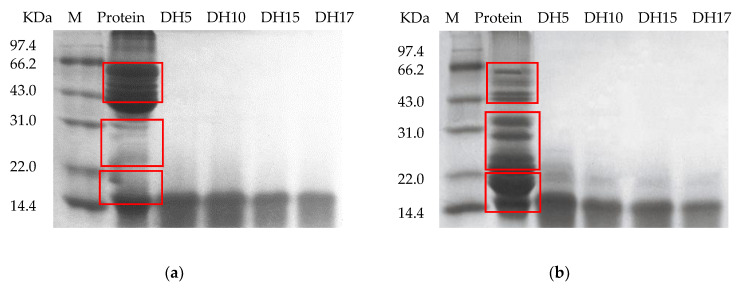
SDS-PAGE profiles of EMO meal protein and hydrolysates under non-reducing (**a**) and reducing (**b**) conditions.

In this study, SDS-PAGE analysis was used to provide information about protein molecular weight under non-reducing and reducing conditions. The results of non-reducing SDS-PAGE showed that the predominant bands of EMO meal protein were distributed at 43–66 KDa, 25–35 KDa, and 14–22 KDa, respectively (shown in Figure 1a). Upon hydrolysis, as DH increased from 5 to 17, the bands at 43–66 KDa gradually disappeared, and the appearance of new bands in <14 KDa range was observed. In other words, the structure of EMO meal protein was obviously changed upon settlement with alcalase, and the EMO meal protein was hydrolyzed into smaller molecular weight proteins.

Under reducing conditions, the molecular weight distribution bands were more visible, and basically similar to that of non-reducing SDS-PAGE profiles (shown in Figure 1b). However, some of the fractions with larger molecular weights were not observed. This phenomenon can be attributed to the fact that the protein aggregates were held together by S-S, while part of them were disrupted by the presence of *β*-Me. It was also observed that some larger molecular weight bands disappeared after enzymatic hydrolysis, along with the appearance of new subunits in the range of 22–31 KDa and <14 KDa range, and only minimal changes were seen in different hydrolysates. The bands between 22 and 31 KDa implied the existence of some acidic subunits of the hydrolysates [18], and the acidic subunits gradually disappeared with the increase of DH. With the complete disappearance of acidic subunits, the profiles of hydrolysates from DH10 to DH17 showed little change. This also intimated that those acidic subunits were more sensitive to alcalase and further hydrolyzed to lower molecular weight molecules. Furthermore, the change to lower molecular mass units could improve their potential native values. For example, García Arteaga et al. (2020) who studied the enzymatic hydrolysis on molecular weight distribution of pea protein, pointed out that the solubility of protein was affected by protein structure and small peptides [6]. Zhang and Romero (2020) reported that the molecular weight ecreases of navy bean protein by alcalase digestion resulted in the increase of emulsifying properties [19].

### 3.2. SHF and S-S Content

**Figure 2:**
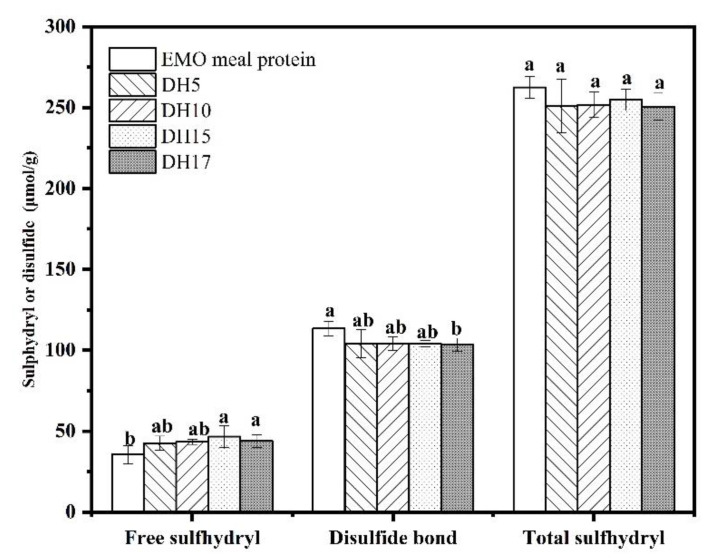
Changes in disulfide bond contents, free sulfhydryl, and total sulfhydryl of EMO meal protein and hydrolysates with different DH. The value of different superscript letters indicated significant difference (*p* < 0.05).

In this study, the analyses of SHT, SHF and S-S were used to study the effect of enzymatic hydrolysis on the internal structure of protein, and the results were shown in Figure 2. The contents of SHT, SHF and S-S in EMO meal protein were 264.27, 35.55, 113.46 μmol/g, respectively. With the increase of DH, no significant difference of SHT contents was observed, while the S-S contents of hydrolysates showed a decreasing trend, and only DH17 (103.34 μmol/g) had showed significant difference compared with the EMO meal protein (113.46 μmol/g). The presence of S-S can stabilize the protein structure [20], while the decrease of S-S content can endow the structure of protein with more flexibility. In addition, the high S-S content is conducive to make protein easily cross-linked and aggregate [21,22], which supported the results of solubility, indicating that the hydrolysates were more soluble than protein.

Corresponding to the gradual decrease of S-S content, a continuing upward trend of SHF content with the increase of DH was also observed from Figure 2. The SHF contents of hydrolysates from DH15 (46.65 μmol/g) to DH17 (43.93 μmol/g) were much higher than the EMO meal protein (35.55 μmol/g), which suggested that the enzymatic treatment unfolded the protein conformation. This phenomenon was also reported by Wen et al. (2019), who applied the ultrasound assisted alcalase to modify watermelon seed protein [23]. These results may be ascribed either to partial unfolding or intramolecular S-S cleavage [24,25]. The changes of SHF and S-S in the hydrolysates further confirmed that EMO meal protein aggregates undergo partial depolymerization during alcalase treatment, which also suggested that the functional properties of the hydrolysates may be improved after alcalase treatment.

### 3.3. Surface Hydrophobicity

**Figure 3:**
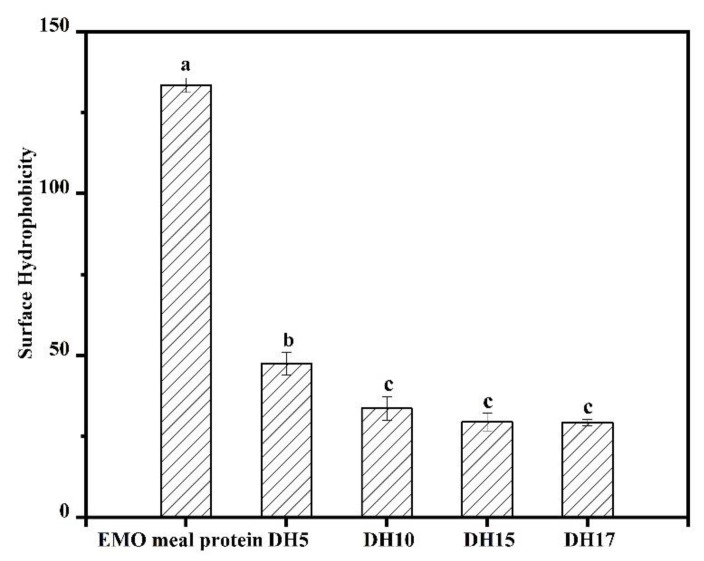
Changes in surface hydrophobicity of EMO meal protein and hydrolysates with different DH. The value of different superscript letters indicated significant difference (*p* < 0.05).

Surface hydrophobicity (H_0_) reflects the behavior and surface properties of protein, which is usually used to characterize the number of hydrophobic groups on the protein’s surface that are in contact with the polar aqueous environment [12]. In this study, H_0_ of the different hydrolysates was found by using a hydrophobic probe (ANS). The results are shown in Figure 3. In contrast to the EMO meal protein (H_0_ = 133.43), the H_0_ of hydrolysates displayed a dramatic reduction as the increase of DH initially, and then a slight decrease from DH10 (H_0_ = 24.33) to DH17 (H_0_ = 20.52). The hydrolysates had low H_0_ because the rigid structure bonded by S-S was broken down by alcalase, resulting in more hydrophilic groups being exposed to the surface of hydrolysates. Similar results were obtained by Shen et al. (2020), who believed that the interaction of hydrophobic groups was enhanced after enzymatic hydrolysis, and then the higher hydrophilicity remained in the solution [26]. This showed that enzymatic hydrolysis could cause the structural transformation of protein from insoluble native aggregates into soluble aggregates. That is to say, the hydrolysates can possess better solubility, which was verified by the results of our experiments. In addition, the higher solubility could unfold the protein structure, which makes the protein rapidly adsorbed on interfaces and improved the other properties, such as emulsion and foamability [21,27].

### 3.4. Intrinsic Fluorescence and Ultraviolet (UV) Spectra

**Figure 4:**
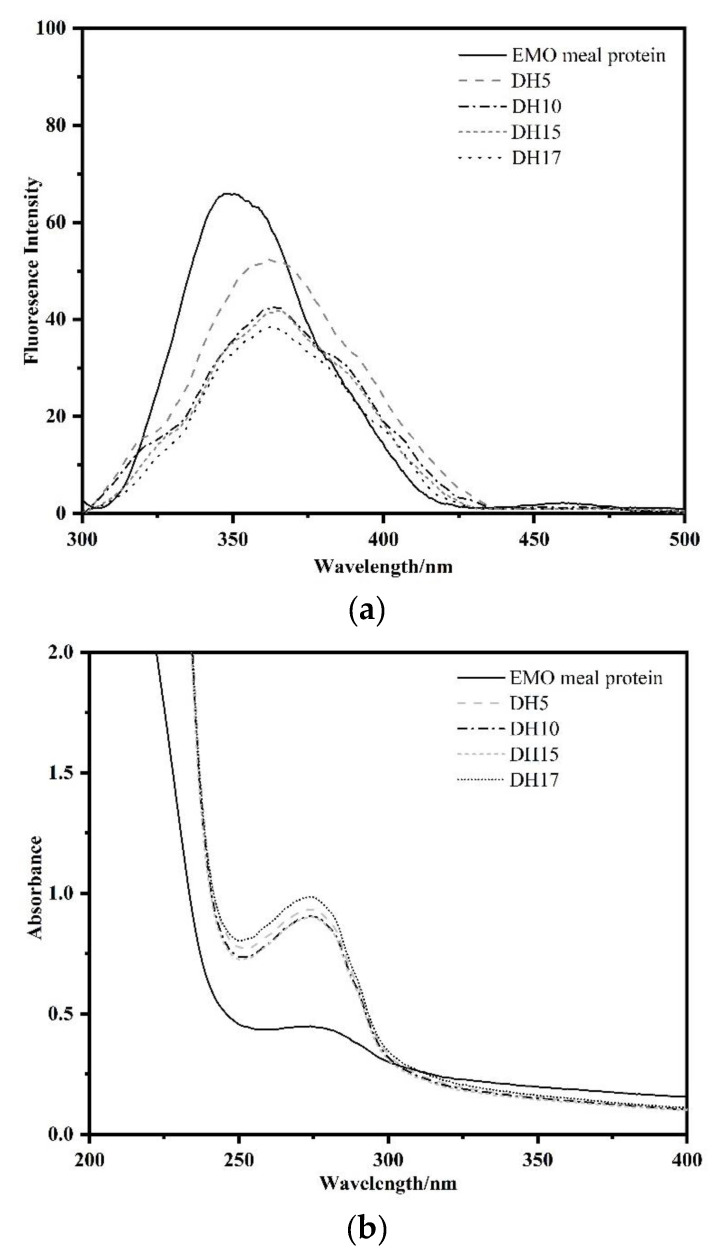
Changes in intrinsic fluorescence (**a**), and ultraviolet spectra (**b**) of EMO meal protein and hydrolysates with different DH.

The intrinsic fluorescence and UV spectra can be used to determine the exposure of protein aromatic amino acids (tryptophan, tyrosine and phenylalanine) to water, and this is linked to changes in protein tertiary conformation [8]. Figure 4a shows the intrinsic fluorescence spectra of various enzymatic hydrolysates. The maximum emission fluorescence wavelength (λ_max_) of EMO meal protein was at 350 nm, suggesting the characteristic fluorescence profile of tryptophan residues. Compared with EMO meal protein, the λ_max_ of hydrolysates exhibited a red-shift to 356.5, 362, 362, 362.5 nm for DH5, DH10, DH15, DH17, respectively, which demonstrated that more aromatic amino acids of EMO meal protein were recognized and hydrolyzed by alcalase. The redshift also indicated that the movement of the tryptophan micro-environment in a hydrophilic direction, and the structure of the hydrolysates was more flexible [2]. In addition, the fluorescence intensity of hydrolysates was weaker than EMO meal protein, and the fluorescence intensity decreased with the increase of DH. According to previous studies, the hydrophobic groups were gradually reburied in the internal structure of the protein via hydrophobic interactions with the increase of DH, resulting in the decrease of fluorescence intensity [8,28,29]. Thus, the downward trend of the fluorescence intensity demonstrated that the hydrophilic groups were gradually exposed to the surface of hydrolysates after alcalase treatment, which was in agreement with the results of H_0_.

The UV spectra of various hydrolysates are shown in Figure 4b. Li et al., (2016) found that the peak wavelengths of phenylalanine, tryptophan and tyrosine were 257, 279, 275 nm, respectively [30]. In the present study, the absorbance intensity of hydrolysate by alcalase was approximately at 273 nm, which showed a blueshift compared with the EMO meal protein. The blueshift may be caused by the destruction of hydrophobic groups and the exposure of chromogenic groups (tryptophan and tyrosine) after alcalase treatment, resulting in an increase of UV spectra in the vicinity of 275 nm. It was also possible that some tryptophan and tyrosine were embedded into the structure of hydrolysate, resulting in the formation of conjugated double bonds, which increased the absorption to near 275 nm.

### 3.5. FTIR Spectrum Analysis

**Figure 5:**
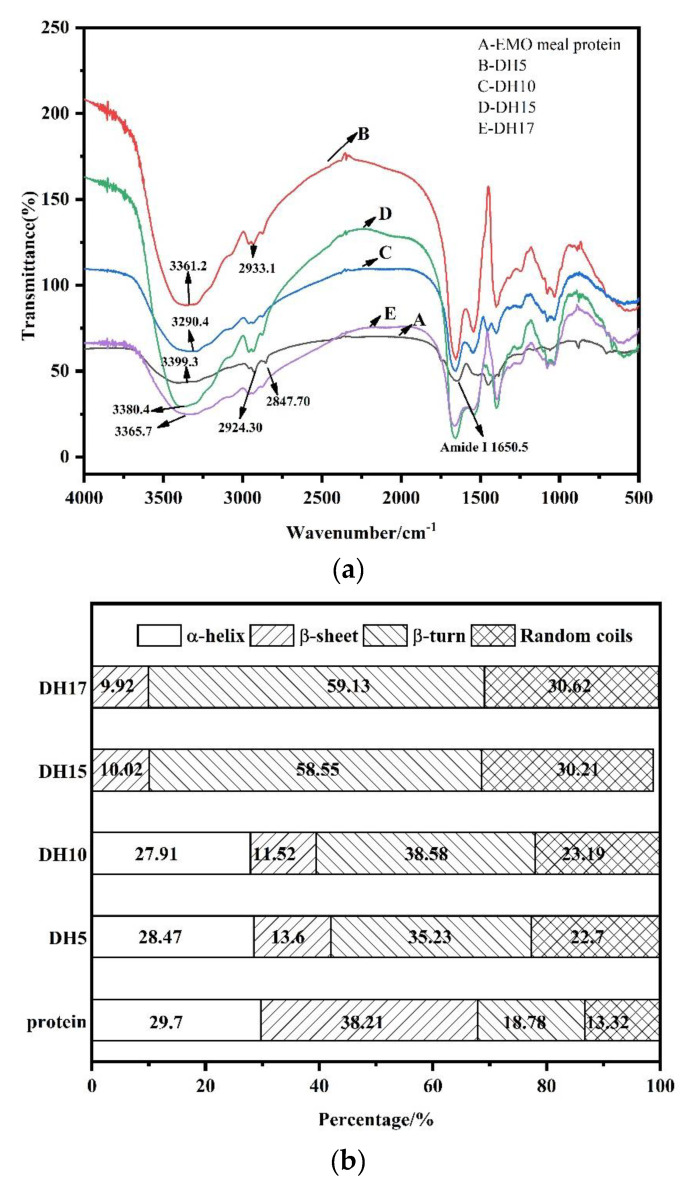
Fourier transform infrared spectra (**a**) and the percentages of structural units (**b**) of EMO meal protein and hydrolysates with different DH.

FTIR spectroscopy is a reliable method for estimating the secondary structure of protein [16]. In the present study, FTIR was used to further examine the physicochemical changes and conformational properties of protein, and the results are shown in Figure 5a. Compared to EMO meal protein, the absorption peak of hydrolysate moved gradually to a lower band with the increase of DH, indicating that the protein structure was changed after alcalase treatment. Previous studies had shown that the characteristic absorption bands at 3200–3400 cm^−1^ and 2800–3000 cm^−1^ were attributed to the stretching vibration of -OH and -CH groups, respectively [31]. All samples had absorption in these bands, and the peaks and peak widths of hydrolysate were weaker than that of protein, implying that the interaction of intramolecular hydrogen bonds in hydrolysate was weakened after enzymatic hydrolysis, which may improve the solubility of hydrolysate.

The amide Ι band formed by the stretching vibration of C=O was mainly distributed at 1600–1700 cm^−1^, and was usually used to approximate the type secondary structure of the protein. The amide Ι band was further divided into four structural units: α-helix (1650–1660 cm^−1^), *β*-fold (1600–1640 cm^−1^), *β*-turn (1660–1700 cm^−1^) and random coil (1640–1650 cm^−1^). The percentages of structural units in the sample were obtained by deconvolution and Gaussian curve fitting, as shown in Figure 5b. Compared with the EMO meal protein, the *α*-helical and β-fold of hydrolysates exhibited a downward trend, while the *β*-turn and random coil exhibited an upward trend. This tendency illustrated that the structure of hydrolysate changed from ordered to disordered. Zhang et al. (2015) reported that the secondary structures of protein was connected by various types of hydrogen bonds. In our study, the alcalase hydrolysis may make the structure of protein more flexible and loose by weakening the intramolecular hydrogen bond [12]. In addition, similar results have been reported by Zheng, Li, & Liu (2020), who claimed that the rearrangement and unfolding of protein during enzymatic hydrolysis leads to the increase of the *β*-turn and random coil content. This structural transformation may lead to better functional properties of the hydrolysates [9]. However, the rigid α-helix in the structure of hydrolysate with DH15 and DH17 was completely hydrolyzed. This indicated that the EMO meal protein was completely transformed into small molecular substance when the DH was greater than 10, which is consistent with the SDS-PAGE profiles. Combined with the decline of the FI and H_0_, we speculate that the hydrophobicity of EMO meal protein was completely destroyed by alcalase, which may break the balance of hydrophilic and hydrophobic groups in protein, resulting in adverse effects on functional properties of hydrolysates.

### 3.6. Analysis of Functional Properties

#### 3.6.1. Solubility

**Figure 6:**
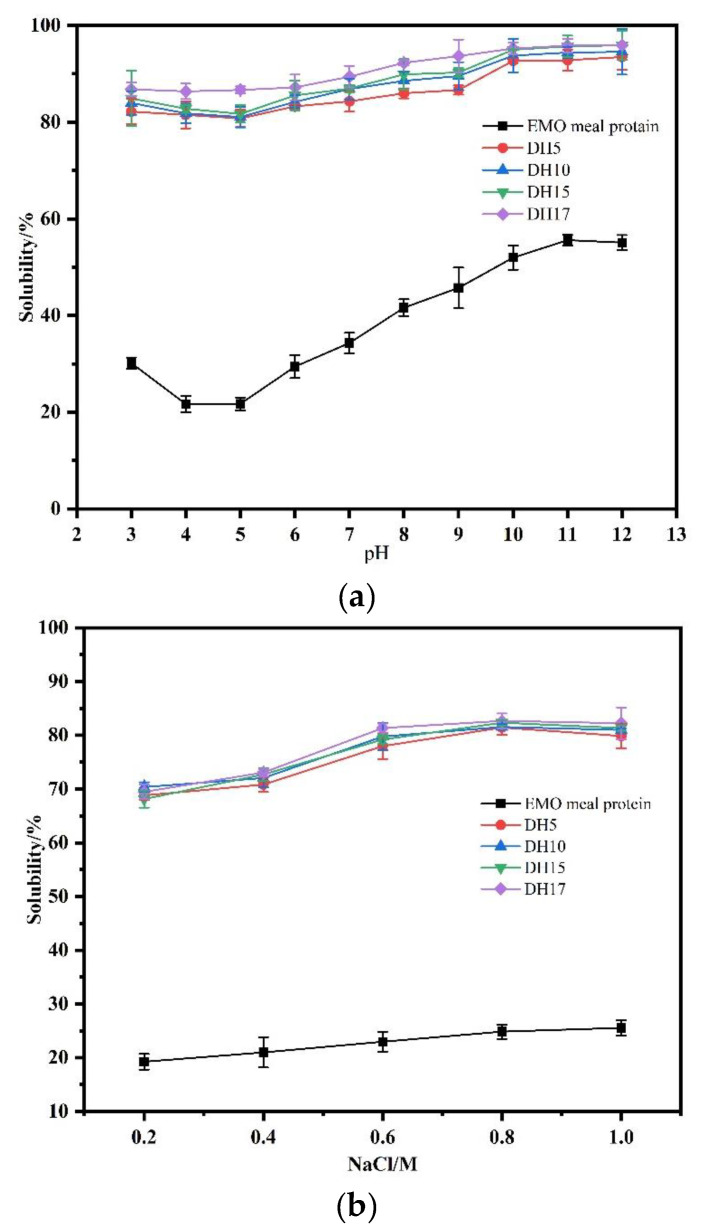
Effect of pH (**a**) and NaCl content (**b**) on the solubility of the EMO meal protein and hydrolysates.

Solubility is a fundamental property of protein, and high solubility aids in the development of other functional properties, such as foamability and emulsifiability. Since proteins are amphoteric polyelectrolytes, the pH value and ionic strength could strongly influence solubility. The influence of pH on the solubility of all samples is shown in Figure 6a. It was found that the EMO protein solubility in the given pH range had a V-shaped curve. The protein solubility was significantly higher at alkaline pH compared to acidic pH, and at pH 4.0, the lowest solubility was observed. That is to say, it was more easily incorporated into neutral or alkaline products as compared to acidic products. After being hydrolyzed by alkaline enzyme, the solubility of the hydrolysates was greatly improved at all pH values, and they also exhibited good solubility in acidic environments. Moreover, the solubility of the hydrolysates slowly increased with increased DH. Considering the changes of structural properties, we found that this increase of solubility was due to the destruction of S-S and hydrophobic groups after alcalase treatment, leading to the increase of hydrophilic groups of protein. Moghadam et al. (2020) reported the same experimental results, indicating that the results could be explained by the release of soluble peptide from the natural insoluble aggregates and the exposure of more amino and carboxyl groups [1]. In other words, the peptide units produced by enzymatic hydrolysis were smaller, more hydrophilic, and more polar.

The solubility of the hydrolysates with different DH in different NaCl concentrations is shown in Figure 6b. It can be seen that the solubility of DH5-DH17 changed slightly as NaCl concentration rose from 0.2 to 0.6 mol/L, then remained stable above 0.6 mol/L. The possible explanation is that the EMO meal protein was hydrolyzed into small molecular peptides, which led to the increase of ion binding sites and repulsion, resulting in greater solubility of hydrolysate. While the NaCl concentration increased to 1 mol/L, the solubility of all samples showed a slight downward trend, which may be due to the salting out effect.

In general, most functional properties of proteins depend on good solubility. In present study, appropriate enzymatic hydrolysis of EMO meal protein could improve solubility, and this change had benefits for applicating in suitable products for food industry and health, such as instant protein powder, protein beverage, nutritional supplements etc.

#### 3.6.2. Water and Oil-Holding Capacities

**Figure 7:**
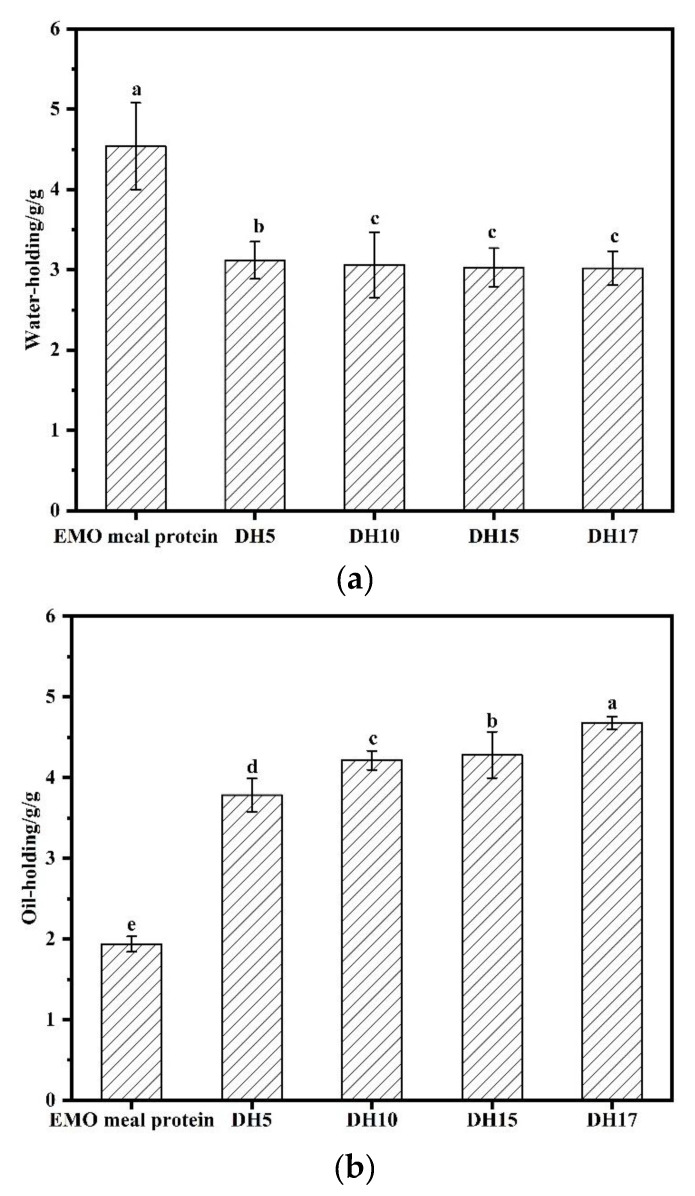
Water-holding capacity (**a**) and oil-holding capacity (**b**) of the EMO meal protein and hydrolysates with different DH. The value of different superscript letters indicated significant difference (*p* < 0.05).

The WHC and OHC of protein are important conditions to improve the food quality as additives. As shown in Figure 7a, EMO meal protein presented the highest WHC (4.55 g/g), while the WHC of the hydrolysates was significantly lower than that of EMO meal protein (mainly distributed in 3.02–3.12 g/g). Similar results were found in the limited hydrolysis of walnut protein by trypsin in Jin et al. (2020), claiming the decrease of WHC after protein hydrolysis [2]. Alcalase treatment exposed hydrophilic groups, increased solubility of protein and decreased the molecular weight. This may not be conducive to forming a network structure between protein and water, which resulted in the reduction of WHC. Combined with the result of FTIR, the structure of hydrolysate was looser and more flexible, which may also affect the WHC ability of hydrolysate.

Compared with EMO meal protein (1.94 g/g), the OHC of hydrolysate showed a rising trend with the increase of DH, and the hydrolysate of DH17 had the highest value of 4.68 g/g (Figure 7b). According to intrinsic fluorescence results, alcalase treatment increased the exposure of the non-polar amino acids in the hydrolysate. Non-polar amino acid chains may have combined with the hydrocarbon chains of fat [32], which enhanced the OHC ability of the hydrolysates. Good oil-holding capacity is considered as a key factor in food application, and it can be widely used in baked products, sausages, or mayonnaise.

#### 3.6.3. Foaming Capacity and Foam Stability

**Figure 8:**
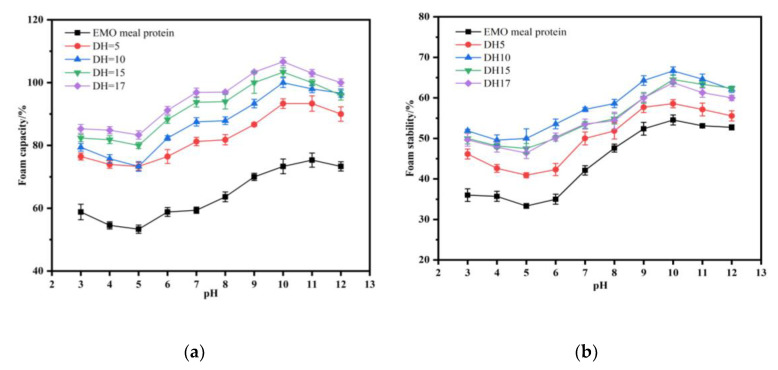
Effect of pH on foam capacity (**a**) and foam stability (**b**) of the EMO meal protein and hydrolysates with different DH.

Protein foam is formed by rearrangement and absorption at the air-water interface during whipping [2]. The effect of different pH values on the foaming properties of hydrolysates is shown in Figure 8a,b. The results indicated that the changes of pH significantly affected FC and FS of the EMO protein and hydrolysates, and the FC and FS of hydrolysates were greater than that of the EMO protein at all given pH values. The minimum value of FC and FS was observed at pH 5.0 for both protein and hydrolysates, the lowest protein solubility was observed at this pH. FC and FS increased greatly when the conditions deviated from pH 5.0, showing the similar trend of solubility change at the range of pH 5.0–12.0, which suggested a strong correlation between foaming properties and solubility. A similar observation was reported by Los, Demiate, Prestes and Lamsal (2020) who investigated the foaming properties of hydrolyzed Carioca bean protein [33]. The foaming characteristics were dependent on the electrical charges on the surface of the proteins, and this also influenced solubility. In the process of increasing pH from 5 to 10, the net charge of protein increased, resulting in the decrease of hydrophobic interaction and increase of solubility. This was conductive to the expansion the protein structure, leading to an increase in the accelerated propagation of protein at the air-water interface, which promoted the improvement of foam formation. In addition, the molecular weight and S-S content of protein were reduced by enzymatic hydrolysis, leading to more flexible peptide chains and a high diffusion rate, which was conducive to the improvement of FC [1]. Moreover, the FC of hydrolysate showed a rising trend with the increase of DH, accompanying the hydrolysate of DH17 had the highest value of 75.33% at pH = 11. However, the maximum FS was observed in DH10 with 66.67% at pH 11, and then decreased with the increase of DH. The small molecular weight and the disappearance of rigid structure made it difficult for the hydrolysate to support the foam strength, resulting in the decrease of FS over DH10. The proteins with stable foamability can serve as a good alternative employed in cakes, beer, bread and tiramisu to improve food quality.

**Figure 9:**
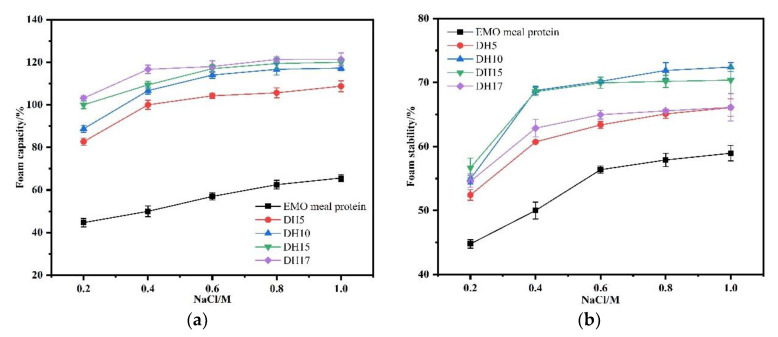
Effect of NaCl concentration on foam capacity (**a**) and foam stability (**b**) of the EMO meal protein and hydrolysates with different DH.

As shown in Figure 9a,b, the effect of NaCl concentration on the foaming properties of hydrolysates were in general similar to that of protein, the FC and FS increased when NaCl concentration increased from 0.2 to 0.6 mol/L, and then showed a slight change higher than 0.6 mol/L. The foaming properties of hydrolysates are significantly higher than that of protein, and the FC rose with increasing DH from 5 to 17. The highest value of FC was observed in DH17, but DH 10 showed a higher value of FS than the other hydrolysates. In fact, the effect of NaCl concentration on foaming properties can be attributed to two aspects: (I) NaCl concentration at low concentration can increase the solubility of protein, resulting in an improvement of foaming properties; (II) the concentration of NaCl concentration continued to increase, resulting in the salting out effect, which broke the protein bound and promoted the precipitation of protein. Therefore, the influence of NaCl concentration on foaming performance was mainly affected by the solubility of protein.

#### 3.6.4. Emulsifying Capacity and Emulsifying Stability

**Figure 10:**
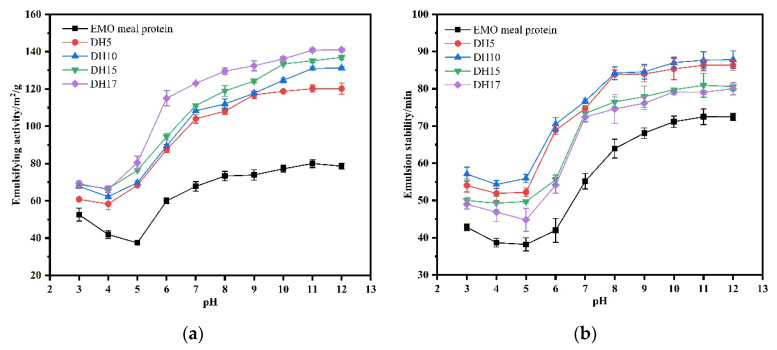
Effect of pH on emulsifying capacity (**a**) and emulsion stability (**b**) of the EMO meal protein and hydrolysates with different DH.

The effects of pH and NaCl concentration on the emulsifying properties of the hydrolysates were demonstrated by EAI and ESI measurements. As shown in Figure 10a,b, the influence of pH on EAI and ESI of the hydrolysates was similar to the results of solubility and foaming properties. After being hydrolyzed by proteases, EAI and ESI were greatly improved at the given pH range. When the pH differed greatly from the isoelectric point, ESI and EAI of all samples gradually increased. Based on the pH-dependent solubility profile, EAI was increased with the increasing of protein solubility, suggesting that the amount of soluble protein was one of the main key factors to the difference of EAI at different pH environments. Furthermore, the EAI of hydrolysates was increased with the increasing of DH. According to the SDS-PAGE mentioned above, it was found that enzymatic hydrolysis produced small molecular peptides, thus reducing the particle size. The protein may have better potential to adsorb at the oil-water interface, when particle size is decreased, leading to the increase of EAI [22]. On the other hand, the solubility of protein was greatly improved after enzymatic hydrolysis, leading to the molecular rearrangement and the improvement of EAI. In addition, the effect of pH on ESI of hydrolysates was the same as that on FS of hydrolysates. The maximum value of hydrolysates at pH = 8 was 84.18% corresponding to DH10, and then decreased with the increase of DH. Avramenko et al. (2013) reported that the emulsifying properties were improved by limited enzymatic hydrolysis, after which a negative effect on emulsification resulted from continued hydrolysis [7]. The decrease could be explained by the formation of a more hydrophilic peptide that associated weakly with the oil-water interface. Another reason could be that the viscoelastic film formed at the interface with a smaller peptide was not able to resist the coalescence of nearby droplets. ESI reflected the balance of hydrophobic and hydrophilic groups of protein. At the initial stage of enzymatic hydrolysis, enzymatic hydrolysis decreased the H_0_, reduced the content of S-S, and increased the solubility, which was beneficial to the balance. However, this balance was broken by further enzymatic hydrolysis. The decrease of H_0_, the destruction of hydrophobic groups and the disappearance of rigid structure decreased the surface activity of hydrolysates, which confirmed our previous conjecture. Therefore, the hydrolysate with DH10 may have a wider application in the food industry, for example the protein hydrolysates can serve as food emulsifier in many food products (such as yoghurt, sausages, beverages or spreads).

**Figure 11:**
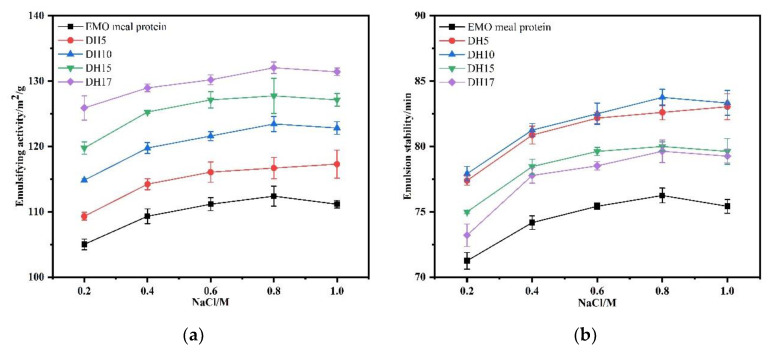
Effect of NaCl on emulsifying capacity (**a**) and emulsion stability (**b**) of the EMO meal protein and hydrolysates with different DH.

As shown in Figure 11a,b, the emulsifying properties of all samples showed a slow increasing trend within the range of 0.2–1.0 mol/L ionic concentration. Within a given range of NaCl concentration, the solubility of protein was enhanced by the electrostatic screening effect of NaCl concentration, resulting in the improvement of the emulsifying properties. In contrast, the highest value of ESI of DH10 was 83.75%, which decreased to 79.63% corresponding to DH17. The interaction of small molecular peptides in salt solution was weak, leading to the decrease of the viscoelasticity of the film. In other words, our results showed that the hydrolysate with DH10 was more salt-tolerant.

## 4. Conclusions

EMO meal protein was hydrolyzed by alcalase to obtain hydrolysates with better functional properties. The experiments showed that the molecular weight, the content of S-S, H_0_ and FI were reduced after alcalase treatment. Then, the structure of protein was more flexible and the surface of the protein was hydrophilic, suggesting that the insoluble aggregates in the protein were transformed into soluble aggregates by alcalase. On the other hand, at the given pH and NaCl concentration, the function properties of hydrolysate were significantly higher than those of the EMO meal protein. The solubility of the hydrolysate was significantly improved, especially in the range of the isoelectric point. It was also found that the hydrolysate with DH10 had the strong surface activities due to the balance of hydrophilic and hydrophobic groups after limited enzymatic hydrolysis. All in all, the hydrolysates obtained by limited hydrolysis of the EMO meal protein, exhibited excellent functional properties, and can be widely used in food and cosmetics as a functional component.

## Data Availability

Data is contained within the article.
